# Supplementary material for: Inhomogeneity in electronic phase and flat band in magnetic kagome metal Co3Sn2S2
Source: Commun Mater. 2025 Nov 3;6(1):235. doi: 10.1038/s43246-025-00939-4 (PMC12583142; doi:10.1038/s43246-025-00939-4)
Supplement: Supplementary file 2 — Supplementary Information [file 43246_2025_939_MOESM2_ESM.pdf]

Inhomogeneity in Electronic Phase and Flat Band in Magnetic Kagome Metal $Co_3Sn_2S_2$

Supplementary Information

Sandy Adhitia Ekahana[1], Satoshi Okamoto[2], Jan Dreiser[1], Loïc Roduit[1], Igor Plokhikh[1], Gawryluk Dariusz Jakub[1], Andrew Hunter[3], Anna Tamai[3], Yona Soh[1*]

1 Paul Scherrer Institute, Forschungstrasse 111, CH-5232, Villigen, Switzerland

2 Materials Science and Technology Division, Oak Ridge National Laboratory, Oak Ridge, Tennessee 37831, USA

3 Department of Quantum Matter Physics, University of Geneva, 24 Quai Ernest-Ansermet, CH-1211, Geneva, Switzerland

*To whom correspondence should be addressed. Email: yona.soh@psi.ch

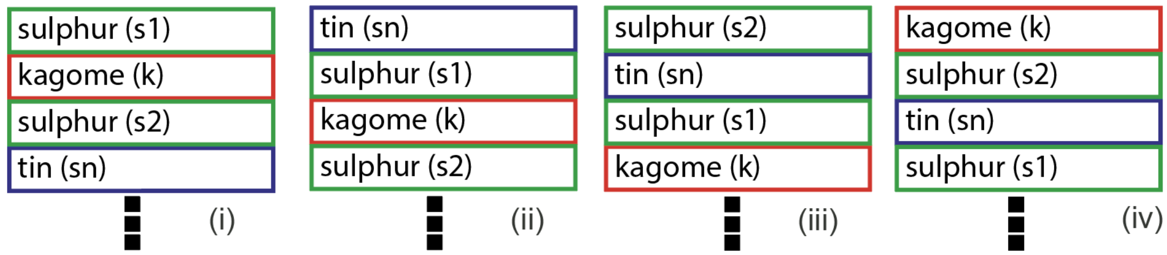

Supplementary Figure 1 Different possible terminations as we cleave the sample. Sn-S1 and Sn-S2 are the most probable cleaving planes, therefore finding (i) and (ii) is more probable than finding (iii) and (iv).

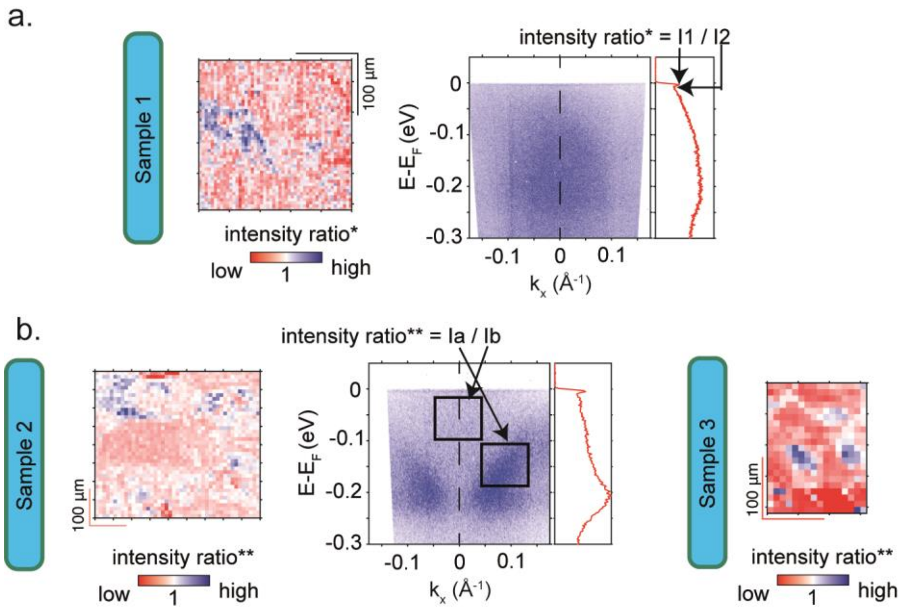

Supplementary Figure 2 Description of intensity ratio * and ** as shown in main Figure 3. (a) Intensity ratio * is obtained by dividing the intensity reading I_1 of the integrated energy distribution curve (EDC) at the flat band position (Fermi level) over the "dip" intensity I_2. The white intensity represents the ratio of one, red represents ratio smaller than one, and blue represents ratio bigger than one. Thus, blue here locates the region with flat band. (b) Intensity ratio ** is obtained by summing the intensity I_a covered by the butterfly wing and dividing it by the summed intensity I_b of the area close to the Fermi level, as shown in the panel. With a similar definition of red-white-blue color, blue here represents the region with the butterfly dispersion.

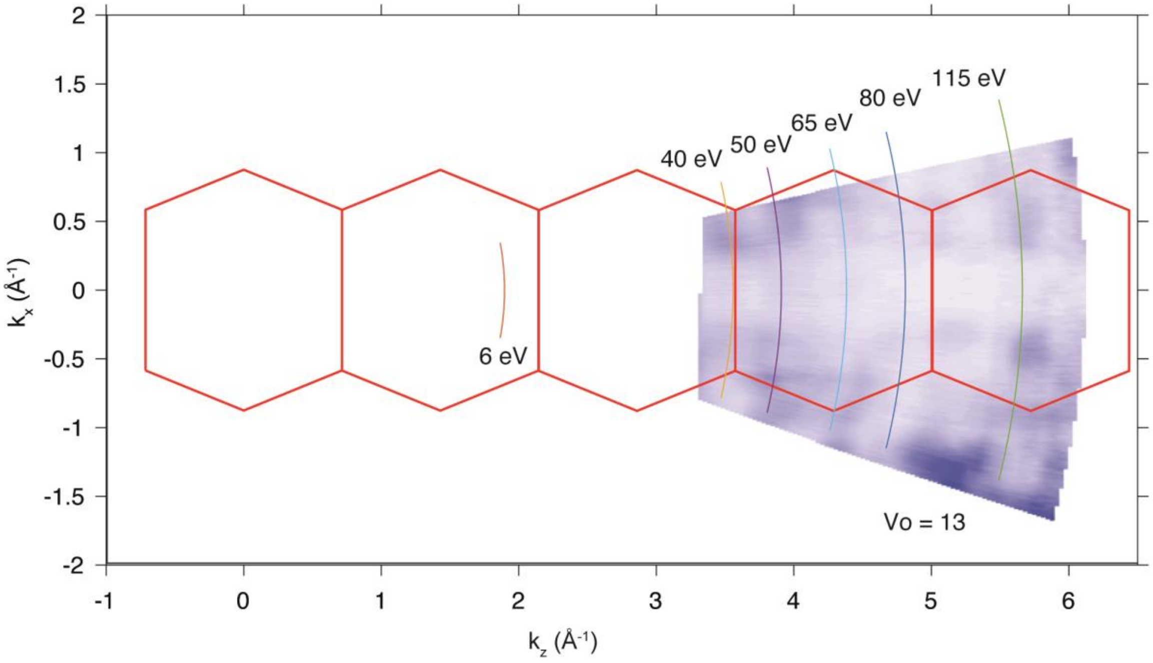

Supplementary Figure 3 Laser of energy 6 eV position at the Brillouin zone given the inner potential V_0 = 13 eV used to transform the photon energy dependent data. Other photon energies are also labeled that signify other k_z positions useful in the discussion. Important is the 80 eV, which lies on the equivalent k_z position as the laser position (shown in main Figure 1**b**).

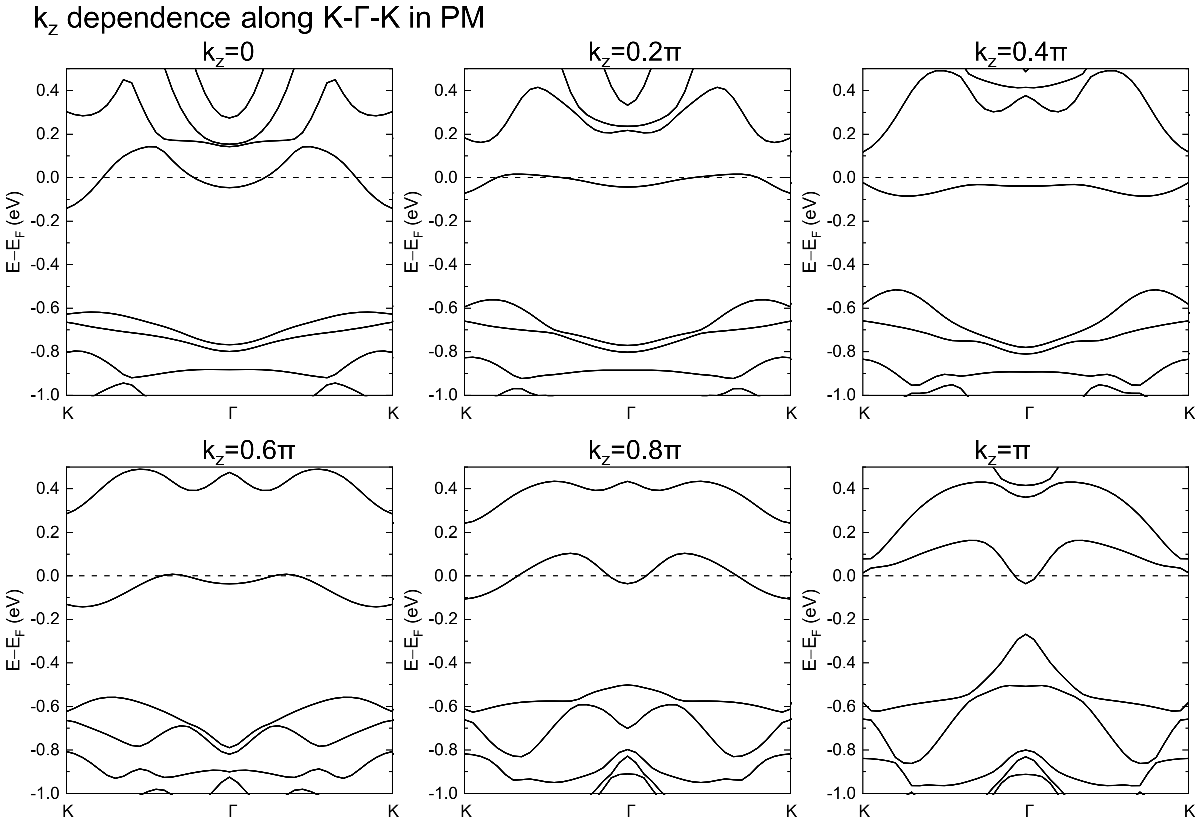

Supplementary Figure 4 Electronic bulk band DFT calculation for the paramagnetic phase at varying k_z positions.

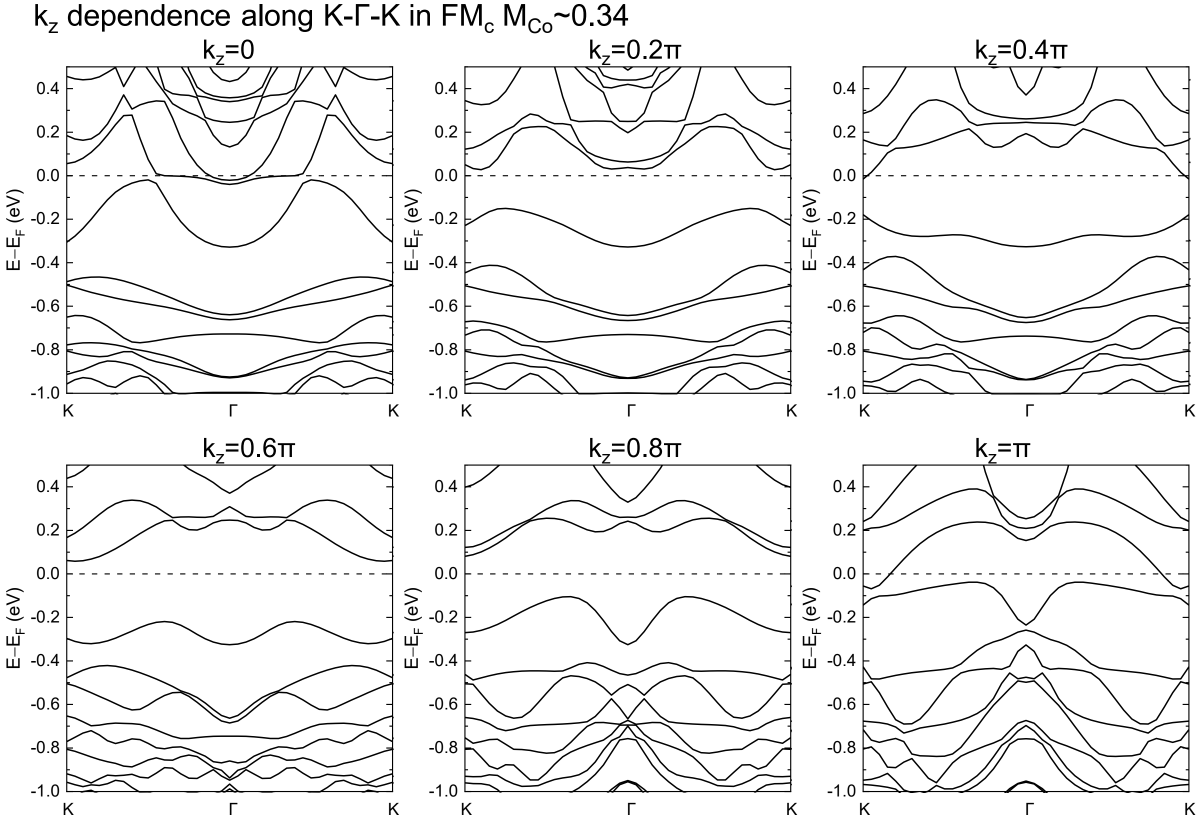

Supplementary Figure 5 Electronic bulk band DFT calculation for the ferromagnetic phase at varying k_z positions with cobalt moment of $\sim 0.34\ \mu_\mathrm{B}$.

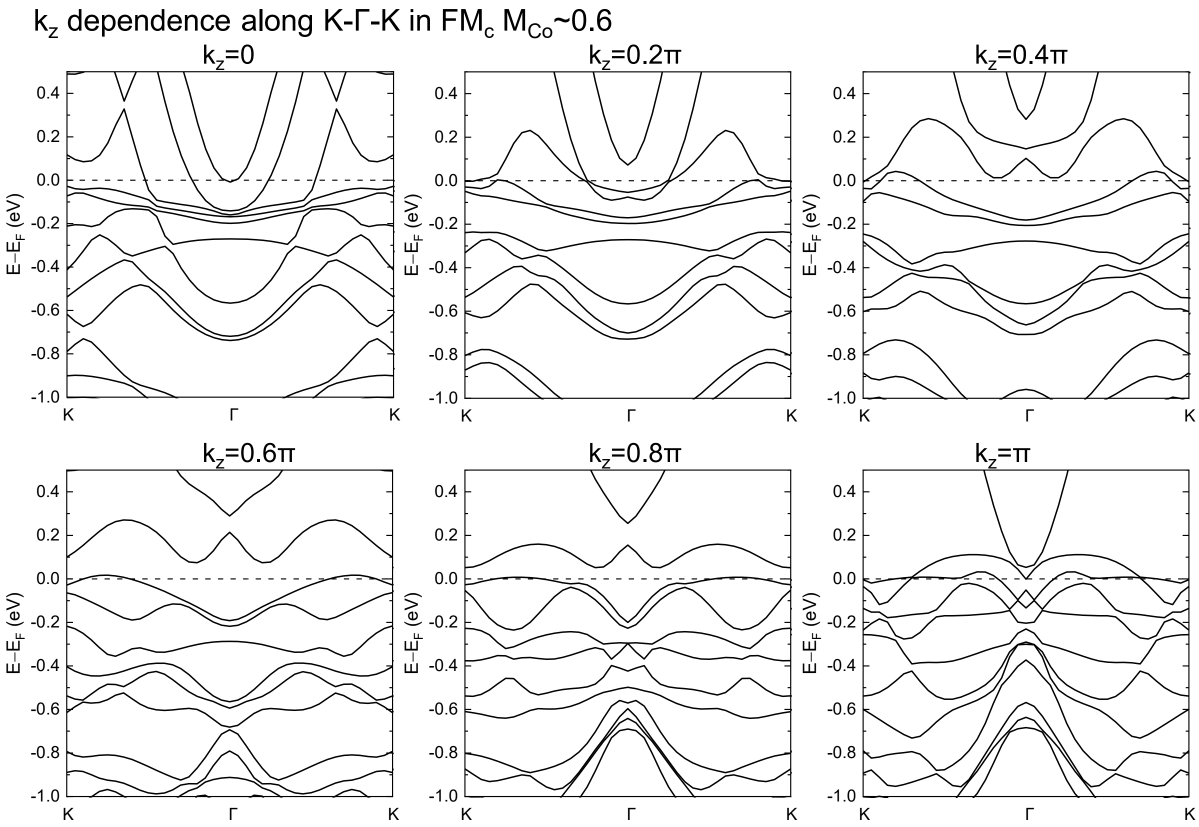

Supplementary Figure 6 Electronic bulk band DFT calculation for the ferromagnetic phase at varying k_z positions with cobalt moment of $\sim 0.6\ \mu_\mathrm{B}$.

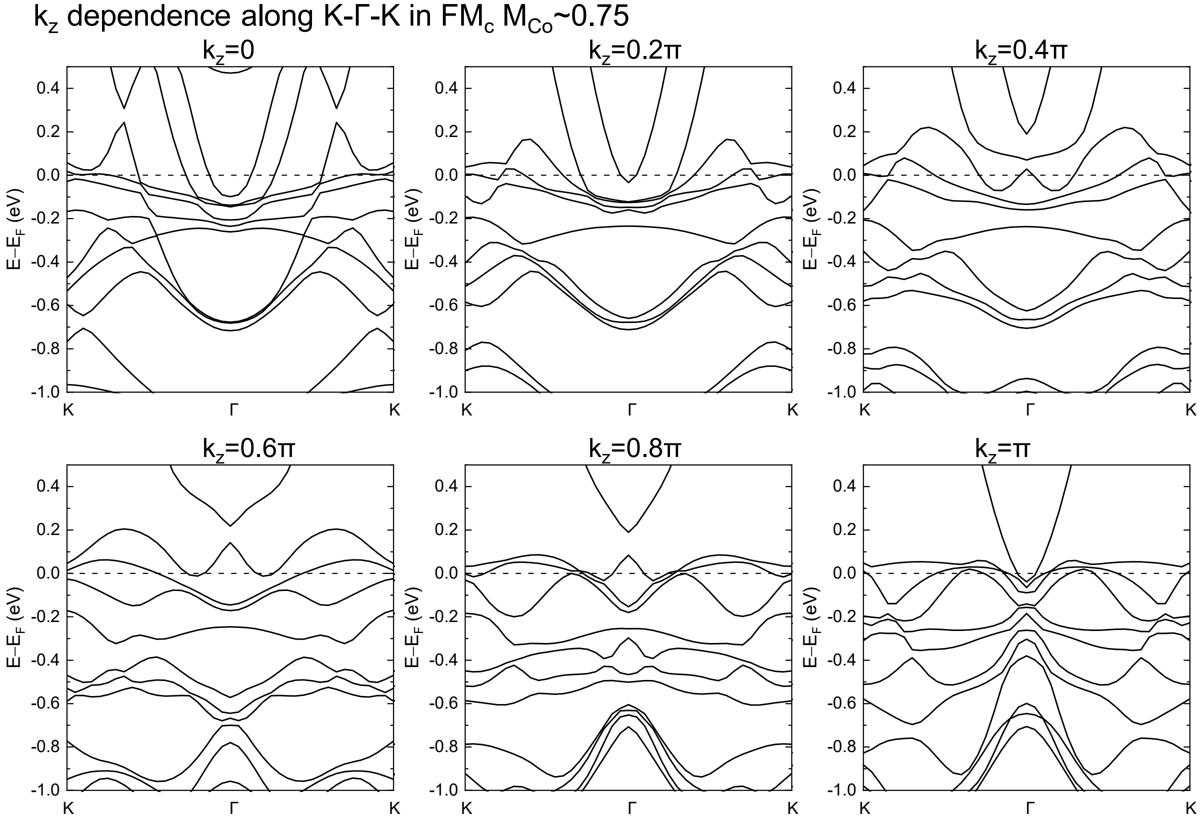

Supplementary Figure 7 Electronic bulk band DFT calculation for the ferromagnetic phase at varying k_z positions with cobalt moment of $\sim 0.75\ \mu_\mathrm{B}$.

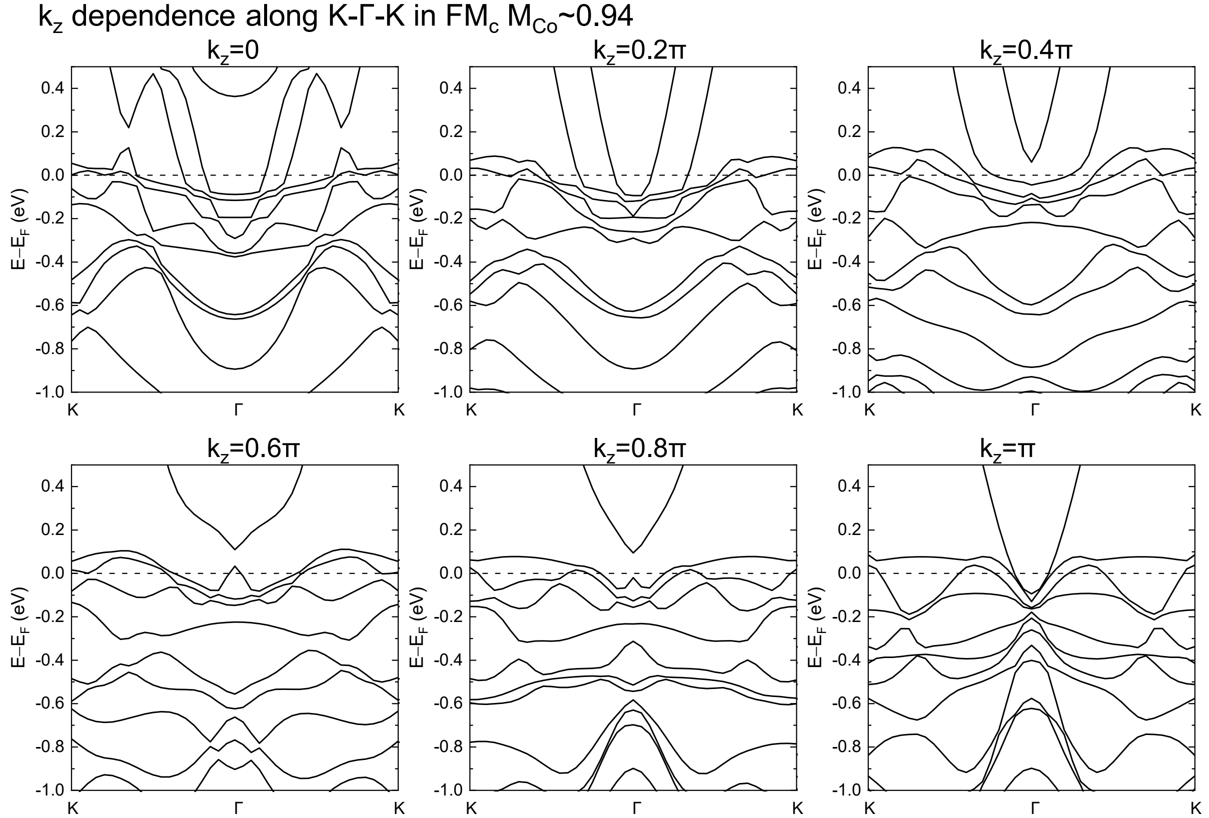

Supplementary Figure 8 Electronic bulk band DFT calculation for the ferromagnetic phase at varying k_z positions with cobalt moment of $\sim 0.94\ \mu_{\mathrm{B}}$.

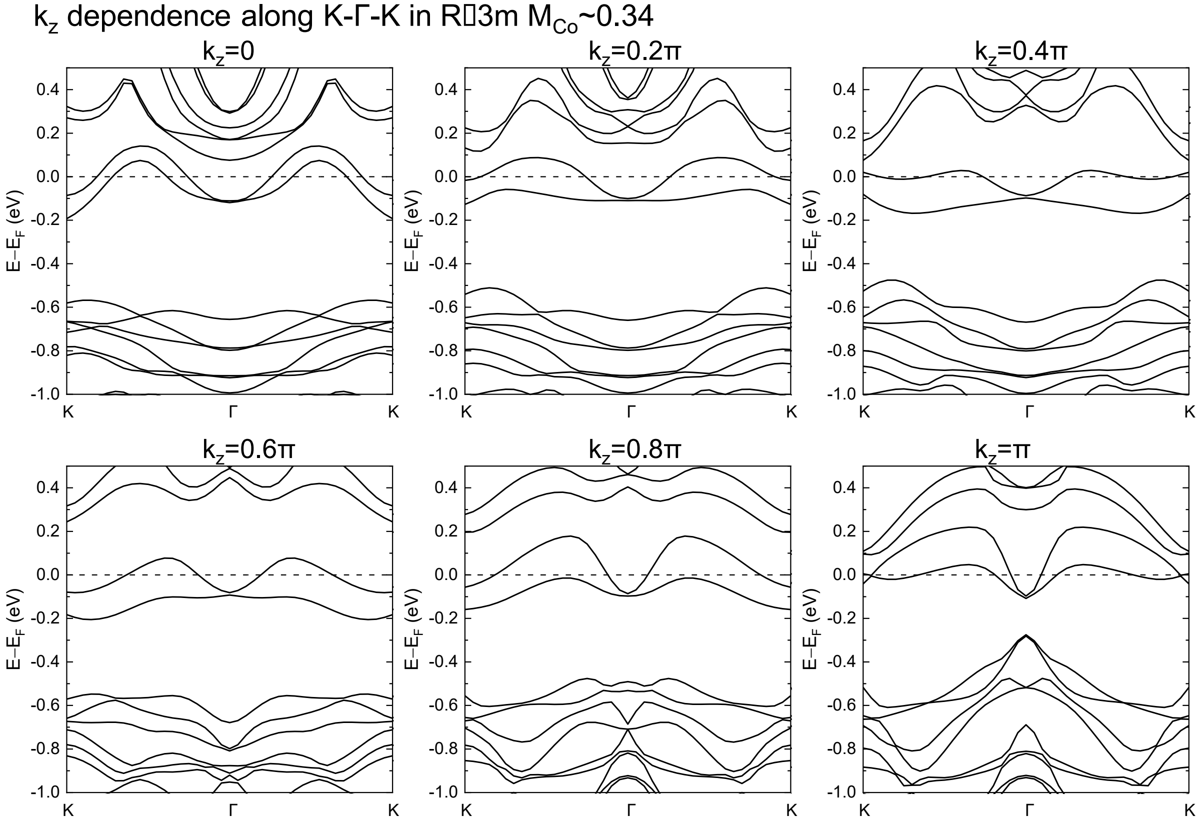

Supplementary Figure 9 Electronic bulk band DFT calculation for the antiferromagnetic phase R-3m (chiral AFM) at varying k_z positions with cobalt moment of $\sim 0.34\ \mu_B$.

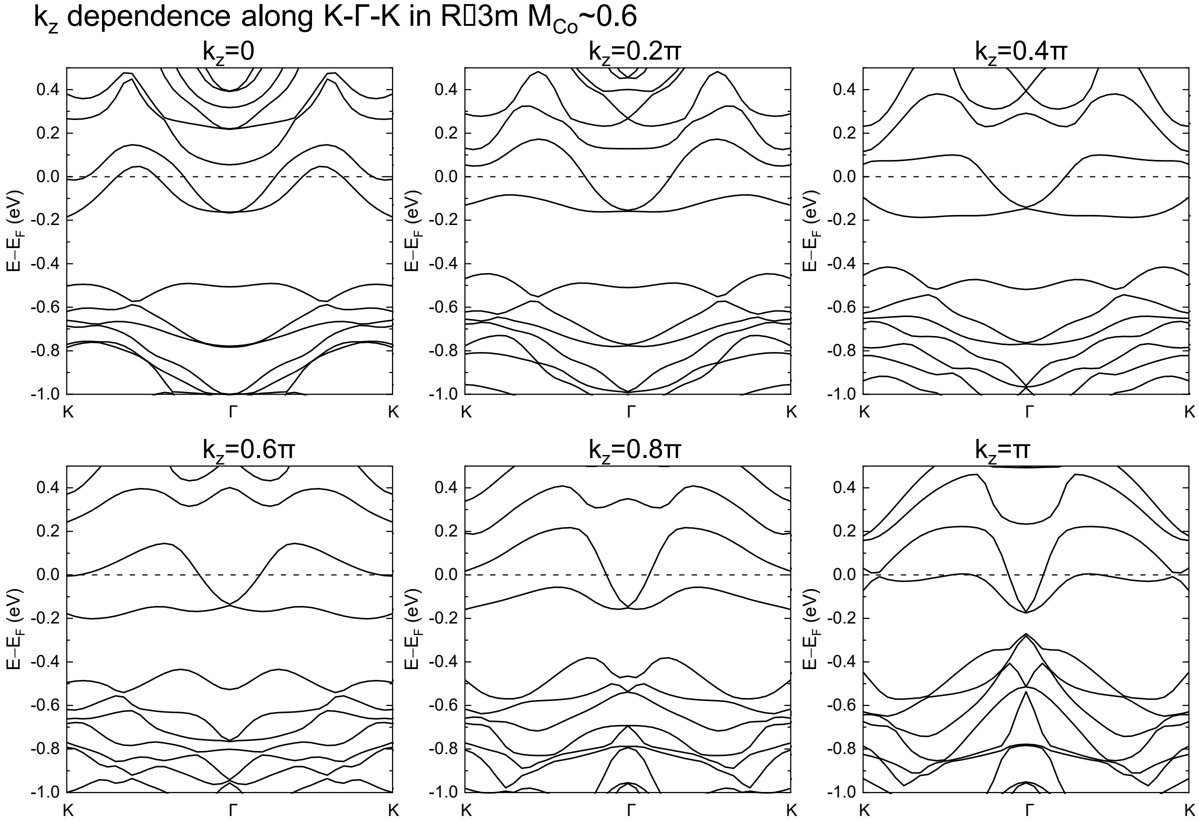

Supplementary Figure 10 Electronic bulk band DFT calculation for the antiferromagnetic phase R-3m (chiral AFM) at varying k_z positions with cobalt moment of $\sim 0.6\ \mu_B$.

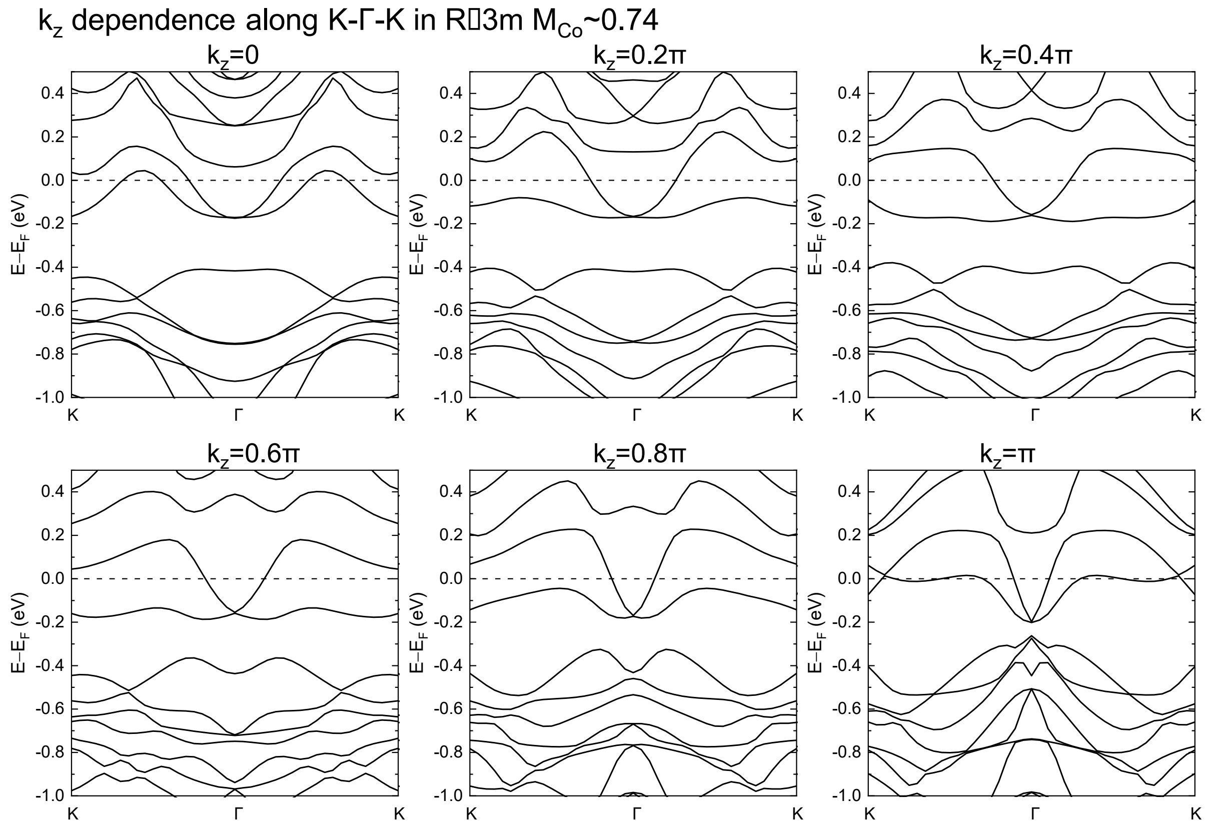

Supplementary Figure 11 Electronic bulk band DFT calculation for the antiferromagnetic phase R-3m (chiral AFM) at varying k_z positions with cobalt moment of $\sim 0.74\ \mu_{\mathrm{B}}$.

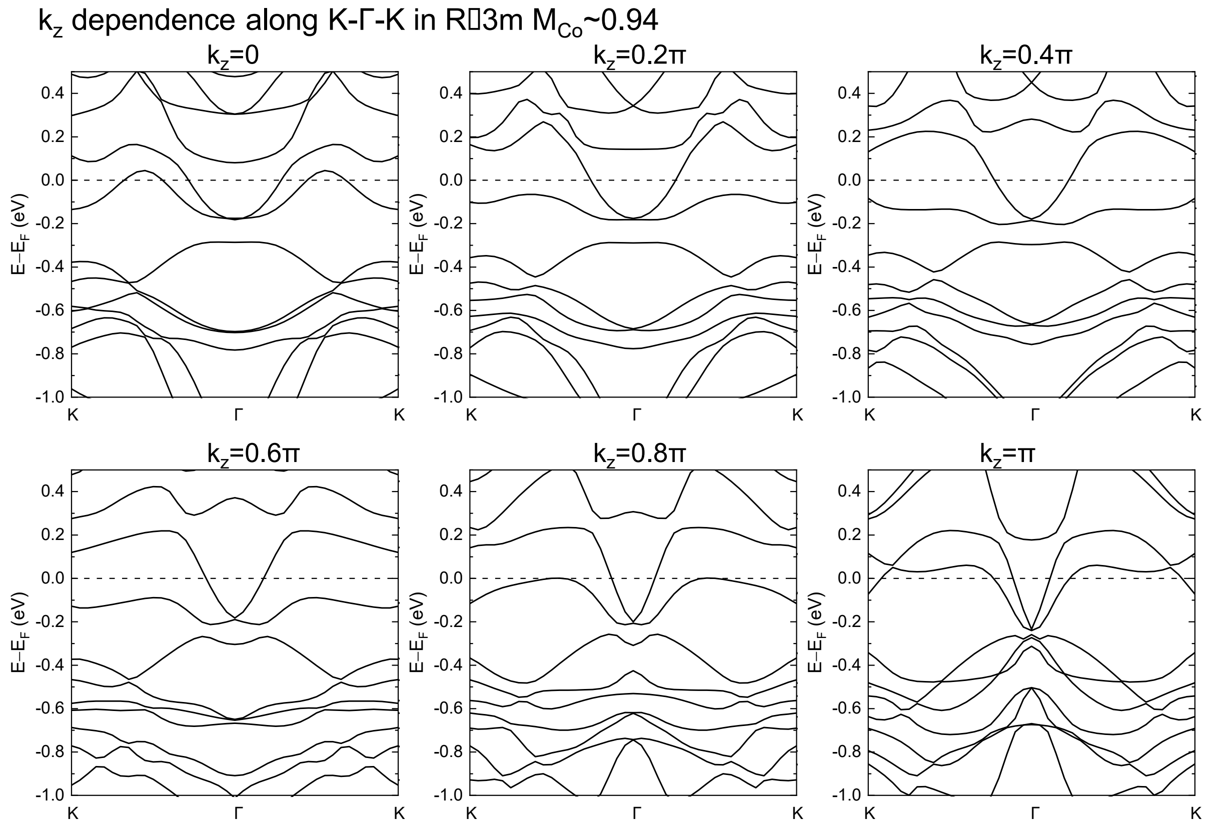

Supplementary Figure 12 Electronic bulk band DFT calculation for the antiferromagnetic phase R-3m (chiral AFM) at varying k_z positions with cobalt moment of $\sim 0.94\ \mu_B$.

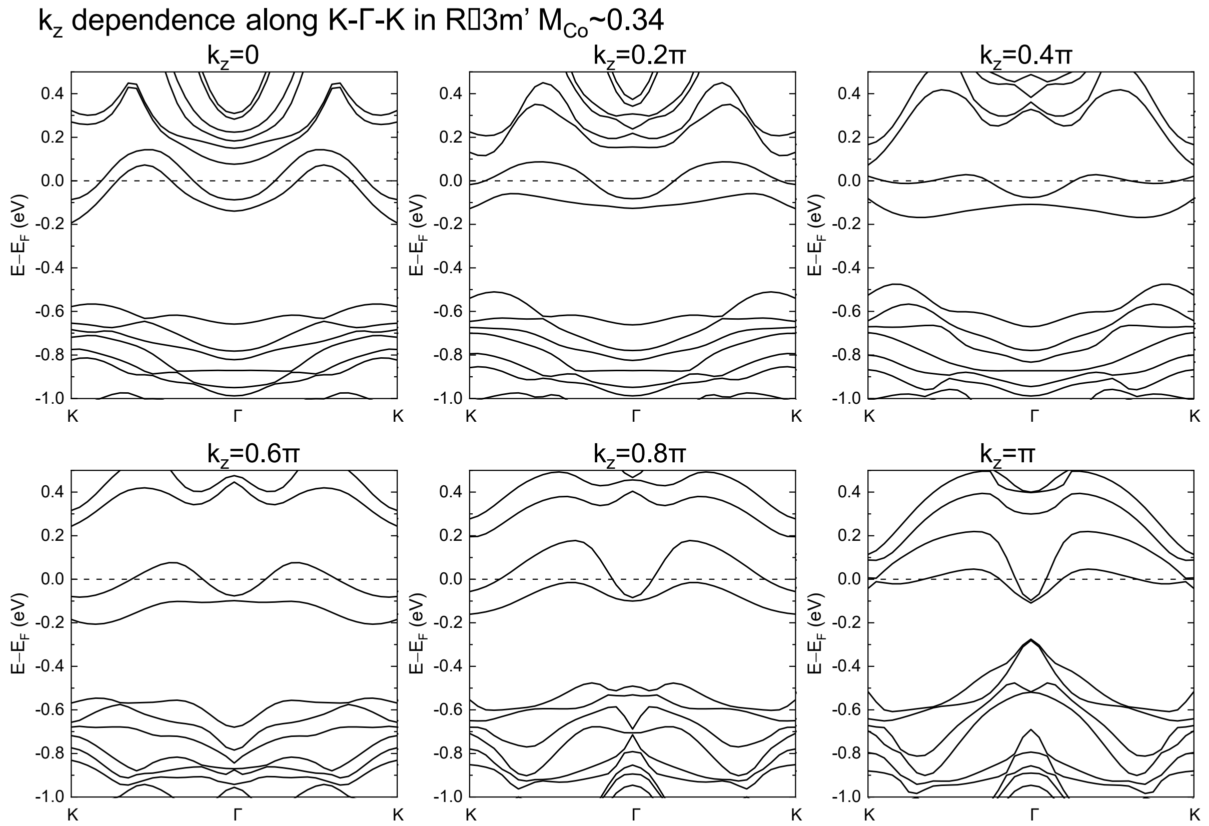

Supplementary Figure 13 Electronic bulk band DFT calculation for the antiferromagnetic phase R-3m' (all in/out AFM) at varying k_z positions with cobalt moment of $\sim 0.34\ \mu_{\mathrm{B}}$.

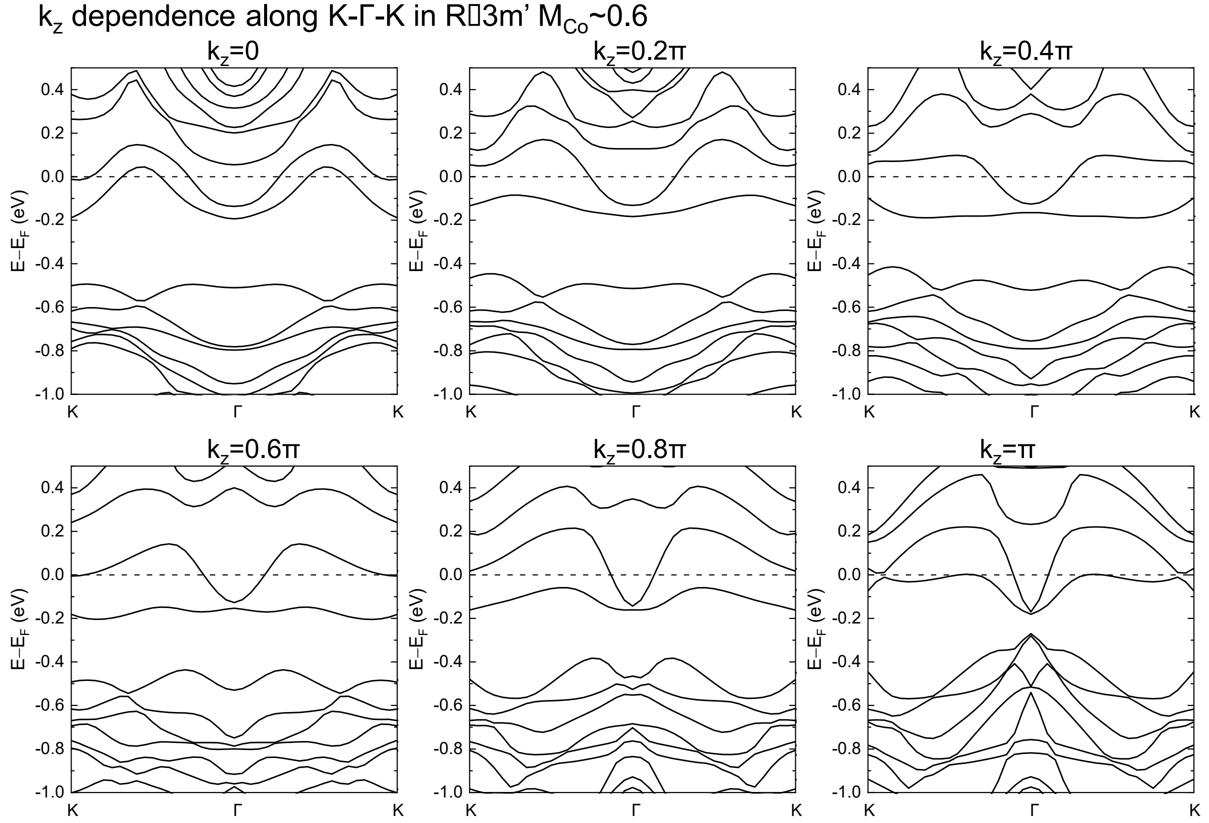

Supplementary Figure 14 Electronic bulk band DFT calculation for the antiferromagnetic phase R-3m'(all in/out AFM) at varying k_z positions with cobalt moment of $\sim 0.6\ \mu_\mathrm{B}$.

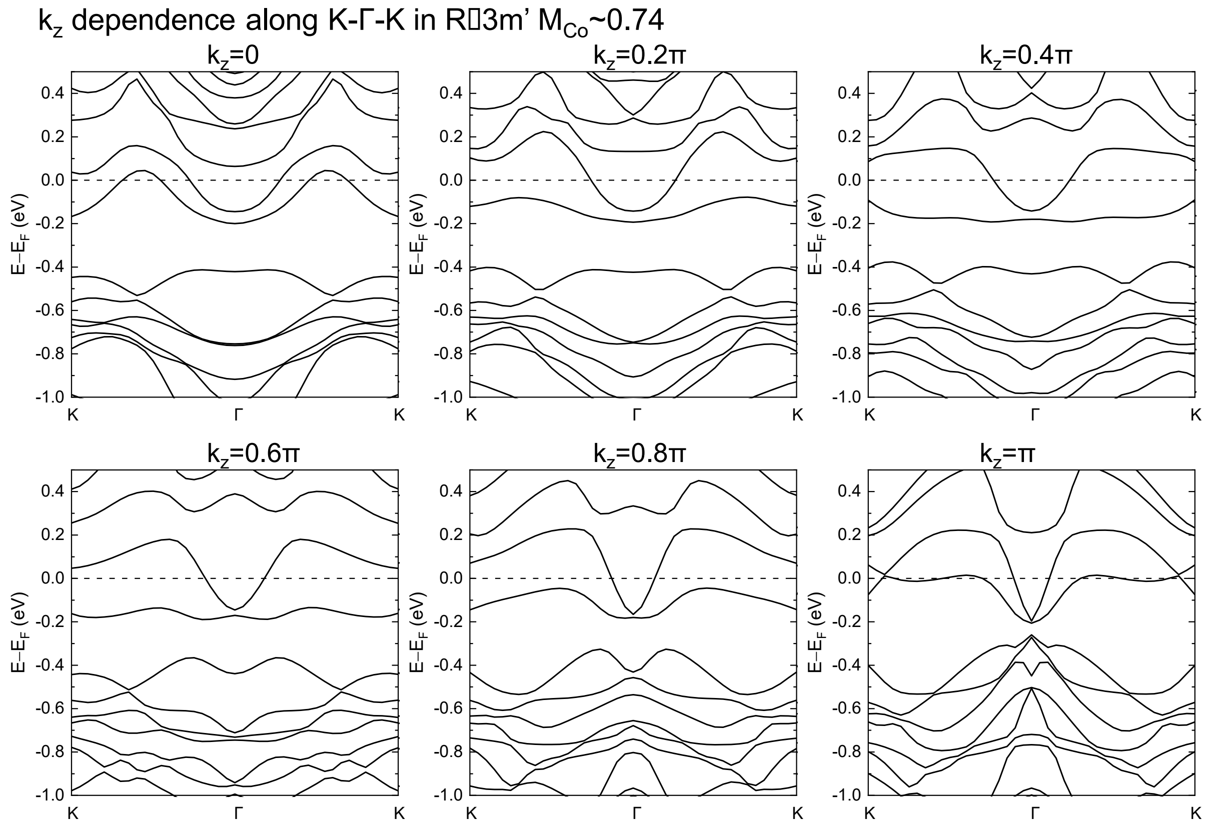

Supplementary Figure 15 Electronic bulk band DFT calculation for the antiferromagnetic phase R-3m’(all in/out AFM) at varying k_z positions with cobalt moment of $\sim 0.74\ \mu_B$.

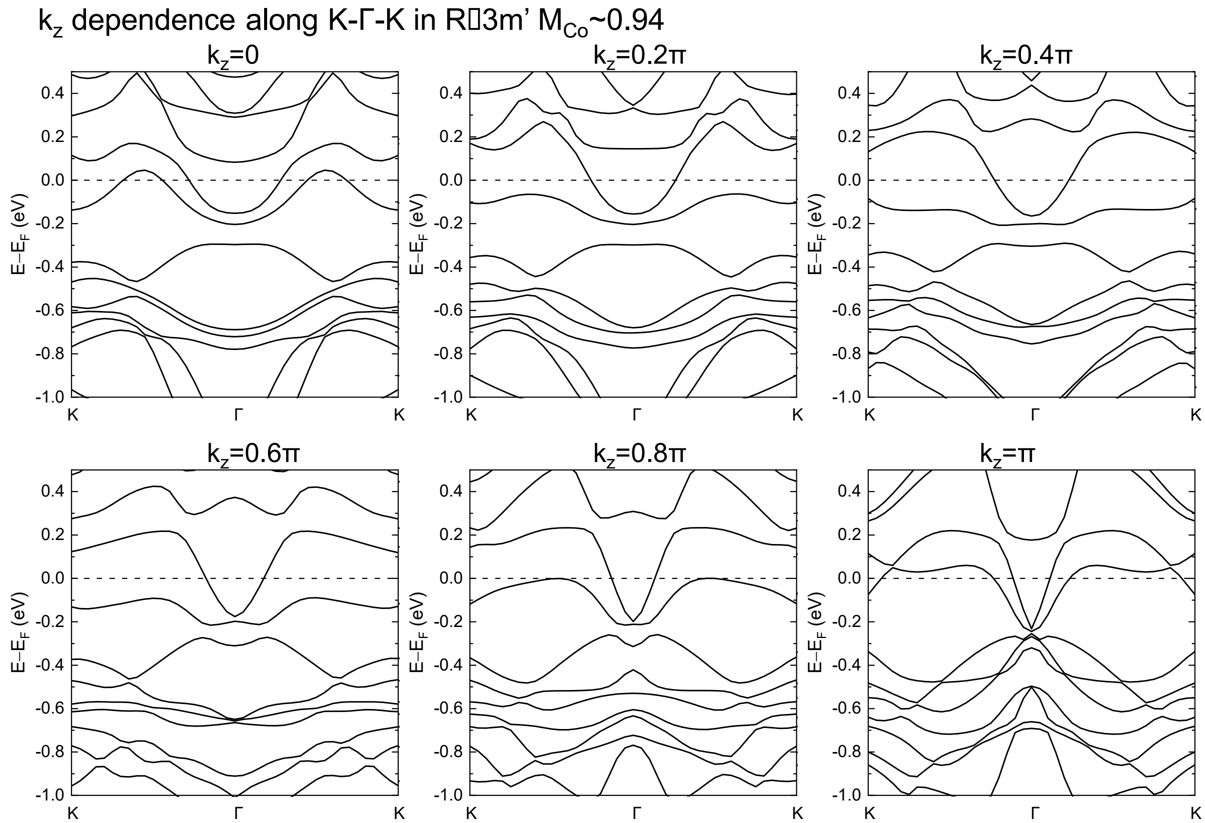

Supplementary Figure 16 Electronic bulk band DFT calculation for the antiferromagnetic phase R-3m' (all in/out AFM) at varying k_z positions with cobalt moment of $\sim 0.94\ \mu_B$.

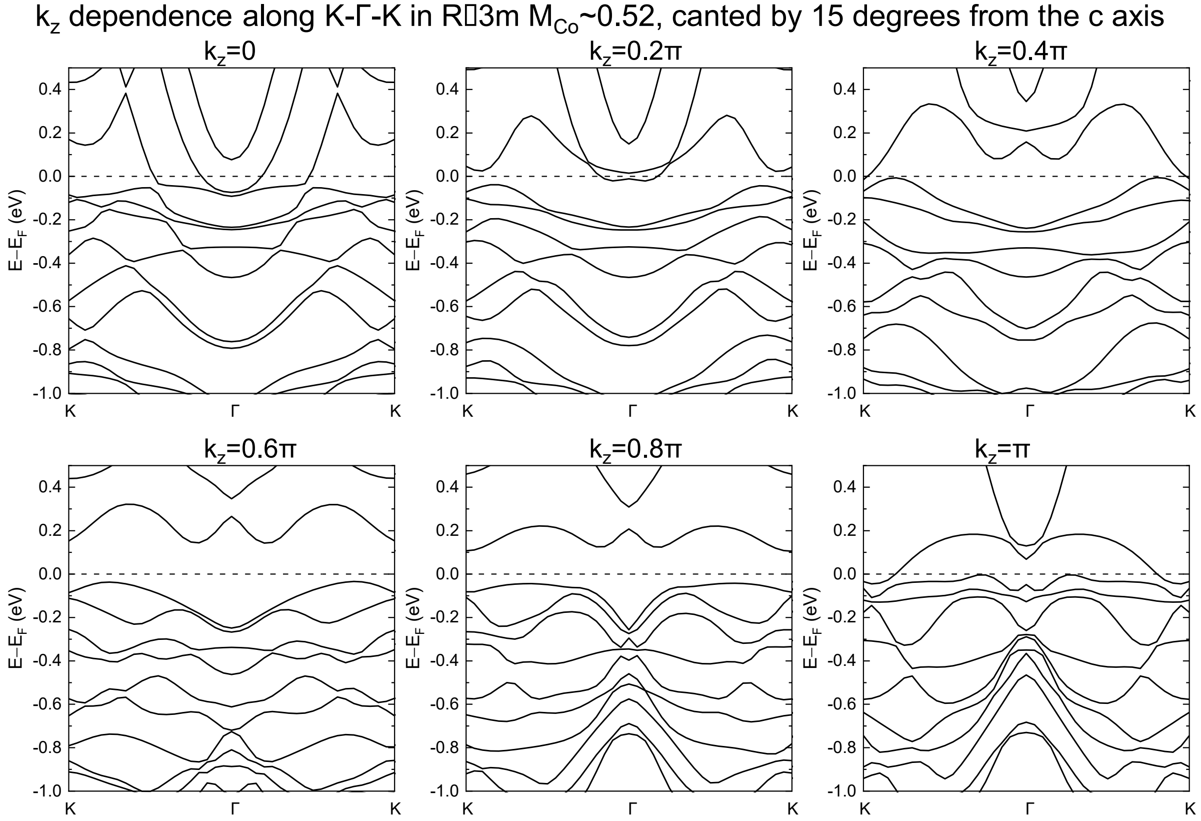

• Difference from a FM phase with similar M_{Co} appears to be very small because of the small canting angle.

Supplementary Figure 17 Electronic bulk band DFT calculation for the combined configuration of ferromagnetic and antiferromagnetic phase R-3m (chiral AFM) such that the moment is canted by 15° from the *c*-axis at varying k_z positions with cobalt moment of $\sim 0.52\ \mu_B$.

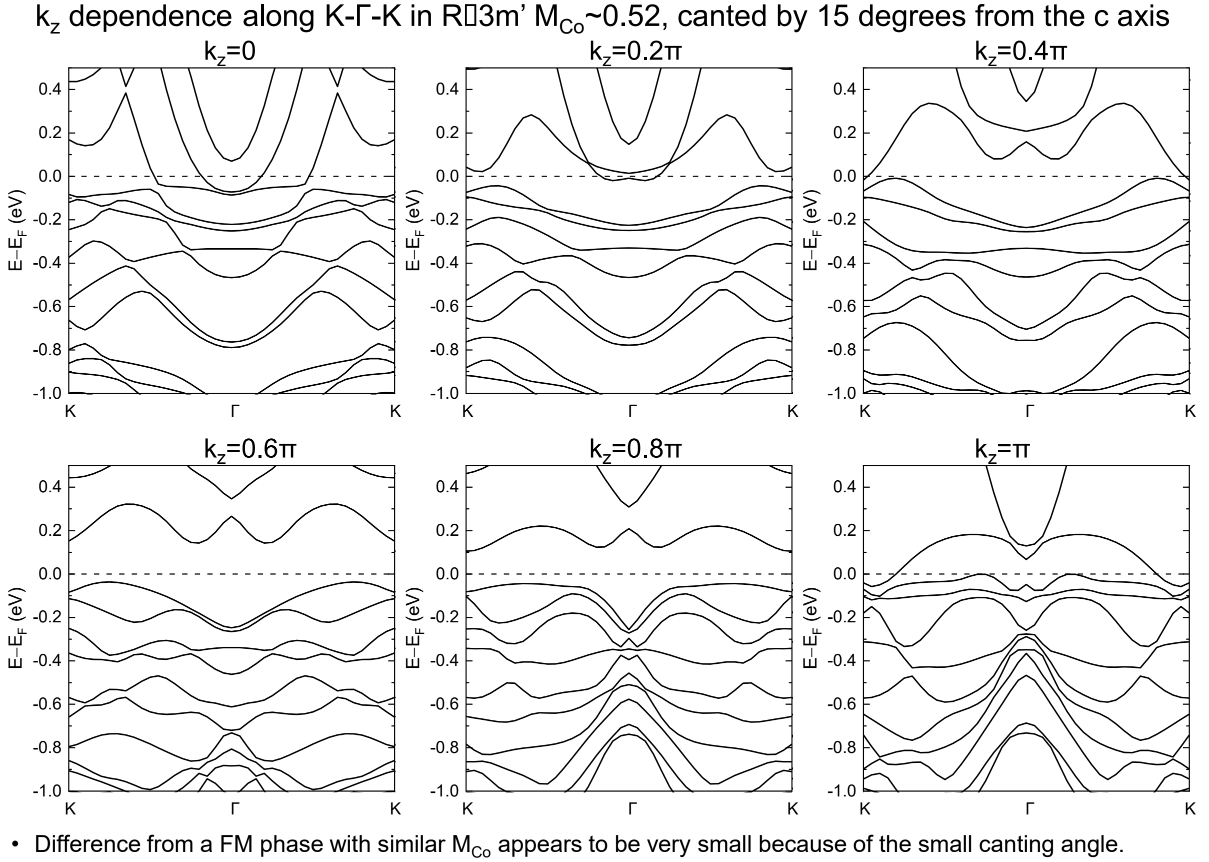

Supplementary Figure 18 Electronic bulk band DFT calculation for the combined configuration of ferromagnetic and antiferromagnetic phase R-3m'(in/out AFM) such that the moment is canted by 15° from the *c*-axis at varying k_z positions with cobalt moment of $\sim 0.52\ \mu_B$.

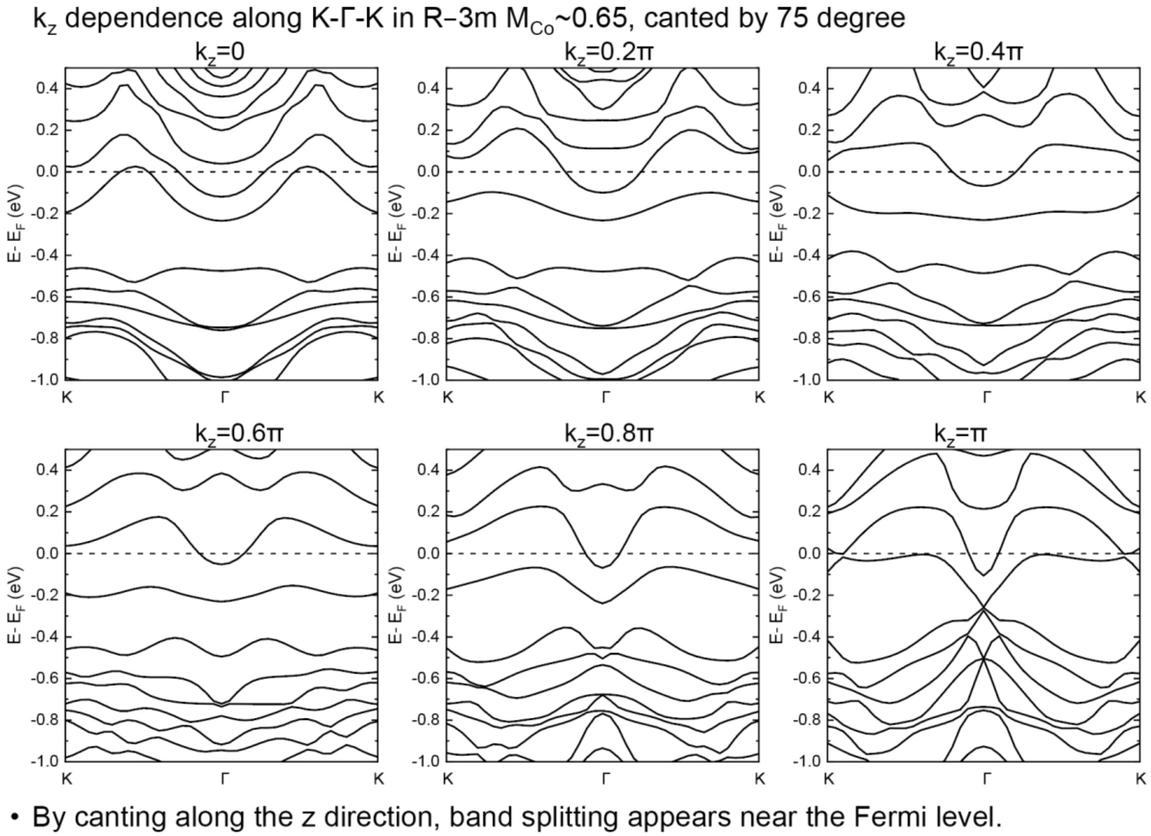

Supplementary Figure 19 Electronic bulk band DFT calculation for the combined configuration of ferromagnetic and antiferromagnetic phase R-3m (chiral AFM) such that the moment is canted by 75° from the *c*-axis at varying k_z positions with cobalt moment of $\sim 0.65\ \mu_\mathrm{B}$.

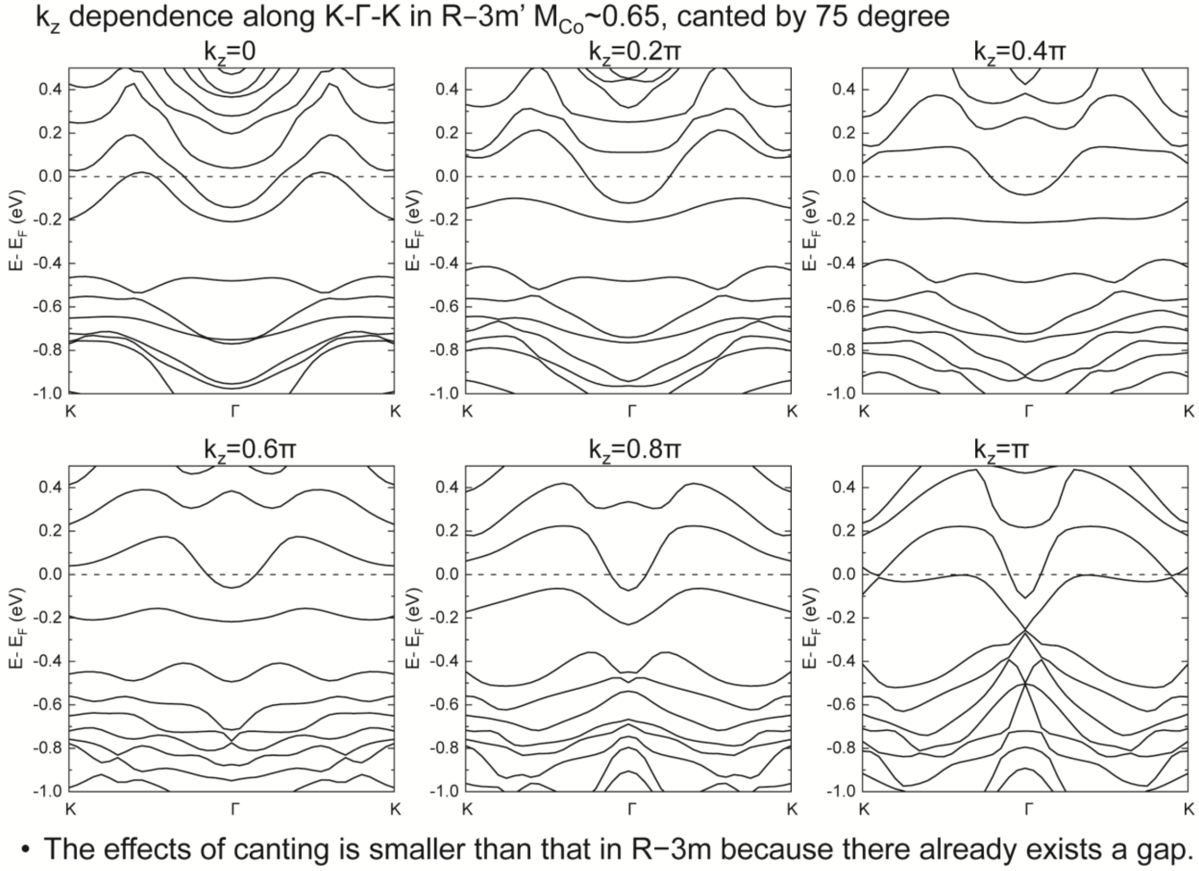

Supplementary Figure 20 Electronic bulk band DFT calculation for the combined configuration of ferromagnetic and antiferromagnetic phase R-3m'(in/out AFM) such that the moment is canted by 75° from the *c*-axis at varying k_z positions with cobalt moment of $\sim 0.65\ \mu_B$.

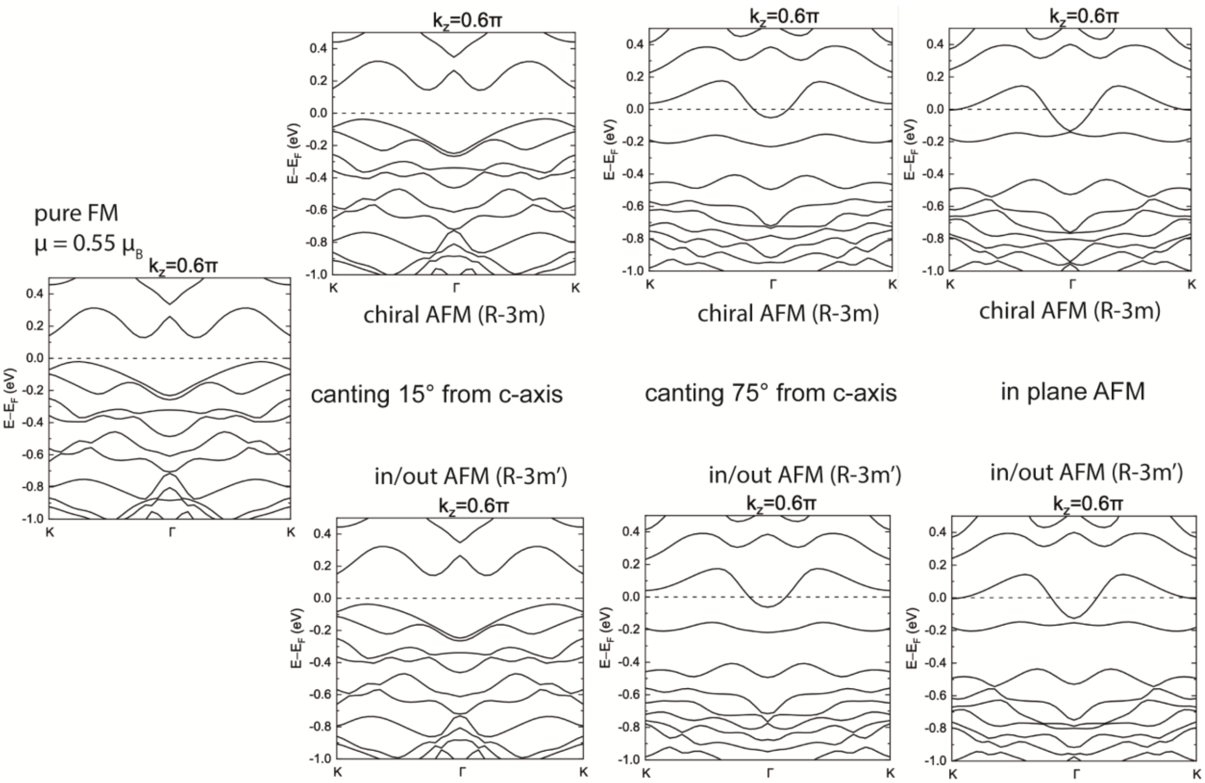

Supplementary Figure 21 Evolution of the electronic band as we rotate the magnetic moment from out of plane (pure FM) into the in-plane AFM mode for both chiral and in/out AFM. We can see even at almost in-plane moment (75° canting), it still does not resemble the butterfly shape that we see. This suggests that the butterfly shape that we see comes from a pure in-plane AFM phase.

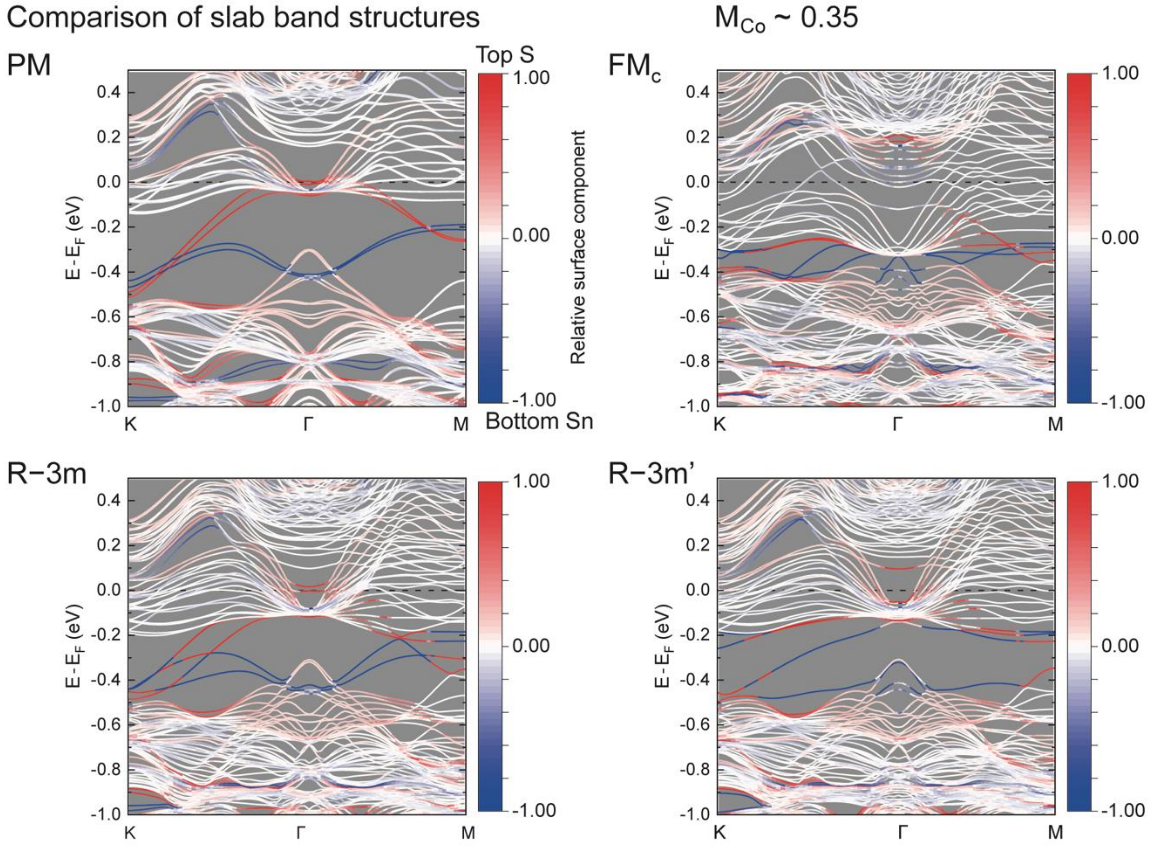

Supplementary Figure 22 Electronic slab band DFT calculation for all the phases: paramagnetic, ferromagnetic, R-3m (chiral AFM), and R-3m'(all in/out AFM) with cobalt moment of $\sim 0.35\ \mu_B$.

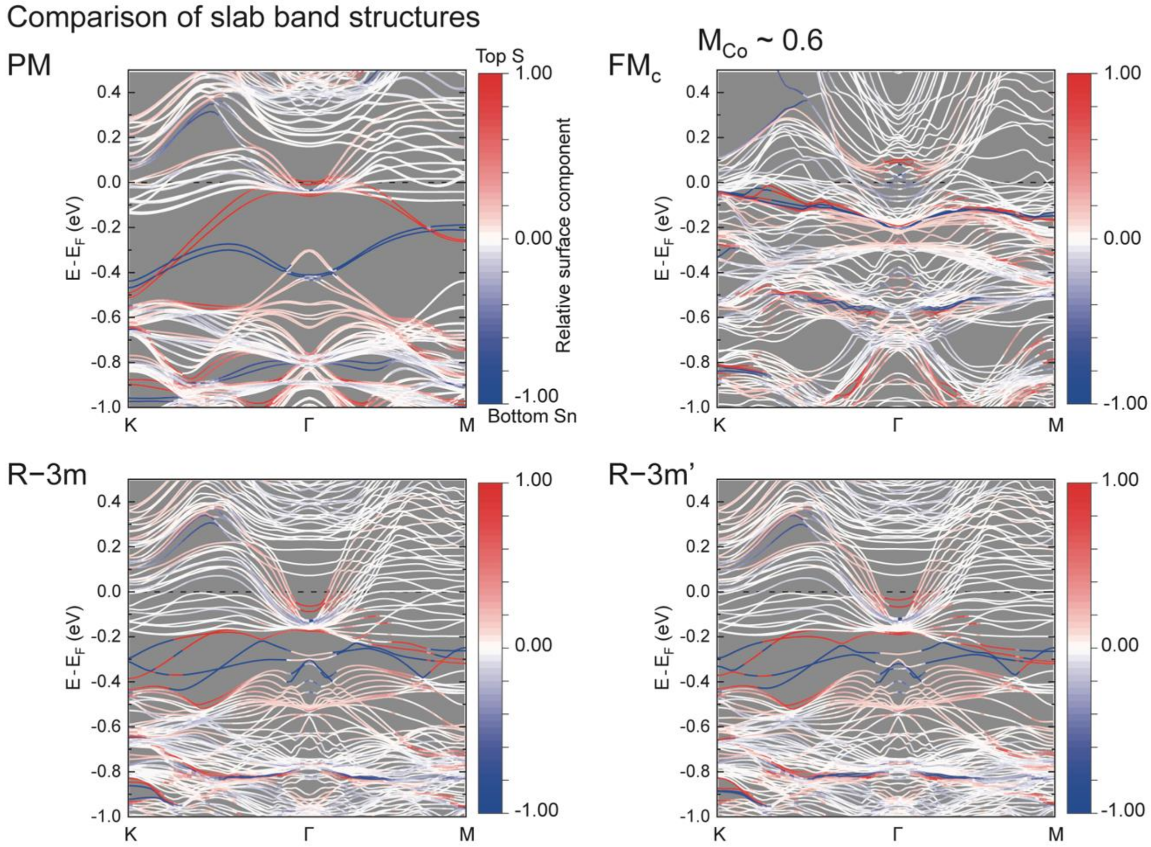

Supplementary Figure 23 Electronic slab band DFT calculation for all the phases: paramagnetic, ferromagnetic, R-3m (chiral AFM), and R-3m' (all in/out AFM) with cobalt moment of $\sim 0.6\ \mu_B$.

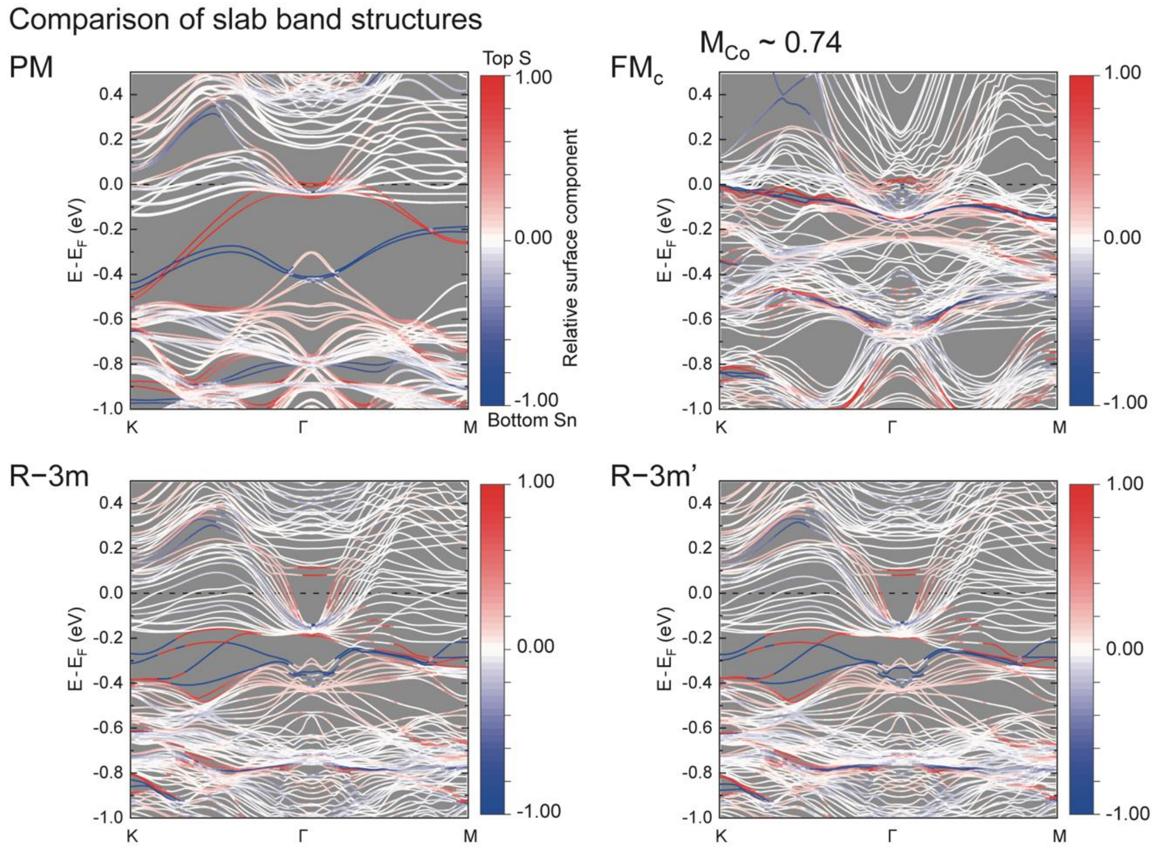

Supplementary Figure 24 Electronic slab band DFT calculation for all the phases: paramagnetic, ferromagnetic, R-3m (chiral AFM), and R-3m’ (all in/out AFM) with cobalt moment of $\sim 0.74\ \mu_B$.

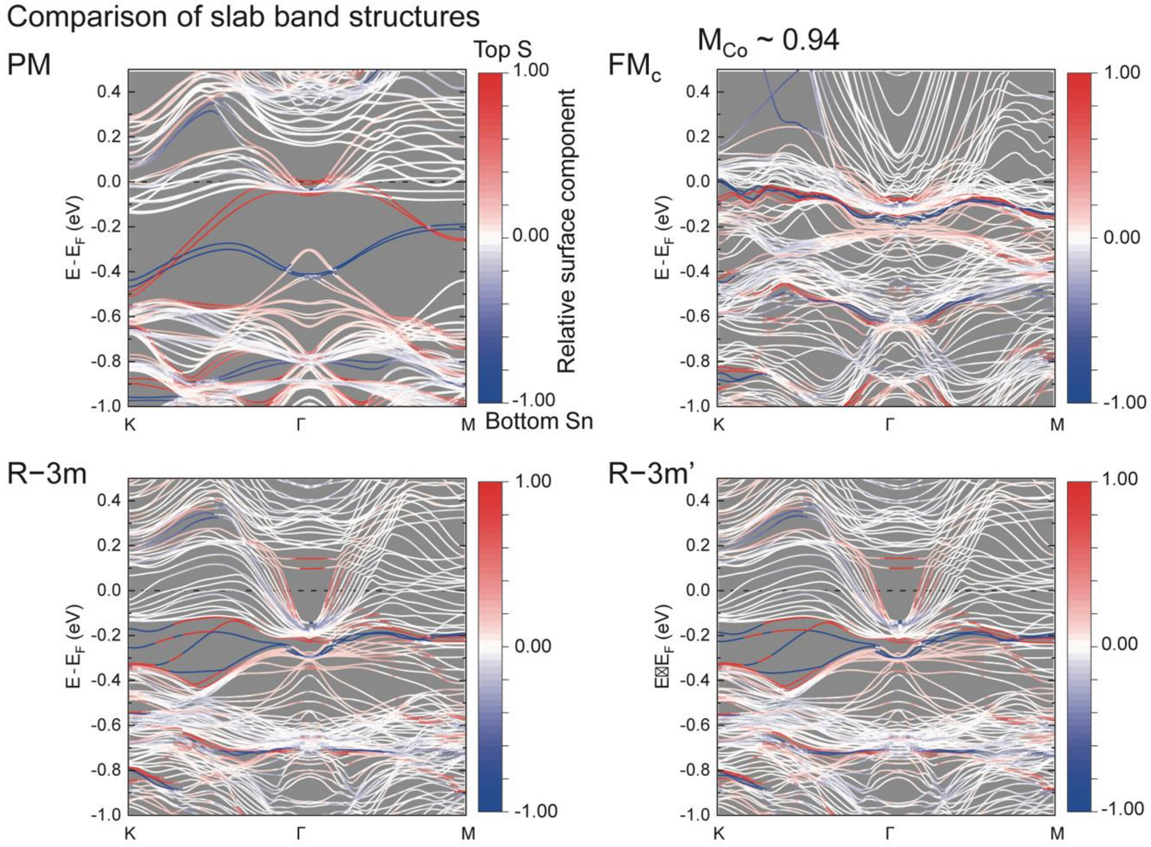

Supplementary Figure 25 Electronic slab band DFT calculation for all the phases: paramagnetic, ferromagnetic, R-3m (chiral AFM), and R-3m' (all in/out AFM) with cobalt moment of $\sim 0.94\ \mu_B$.

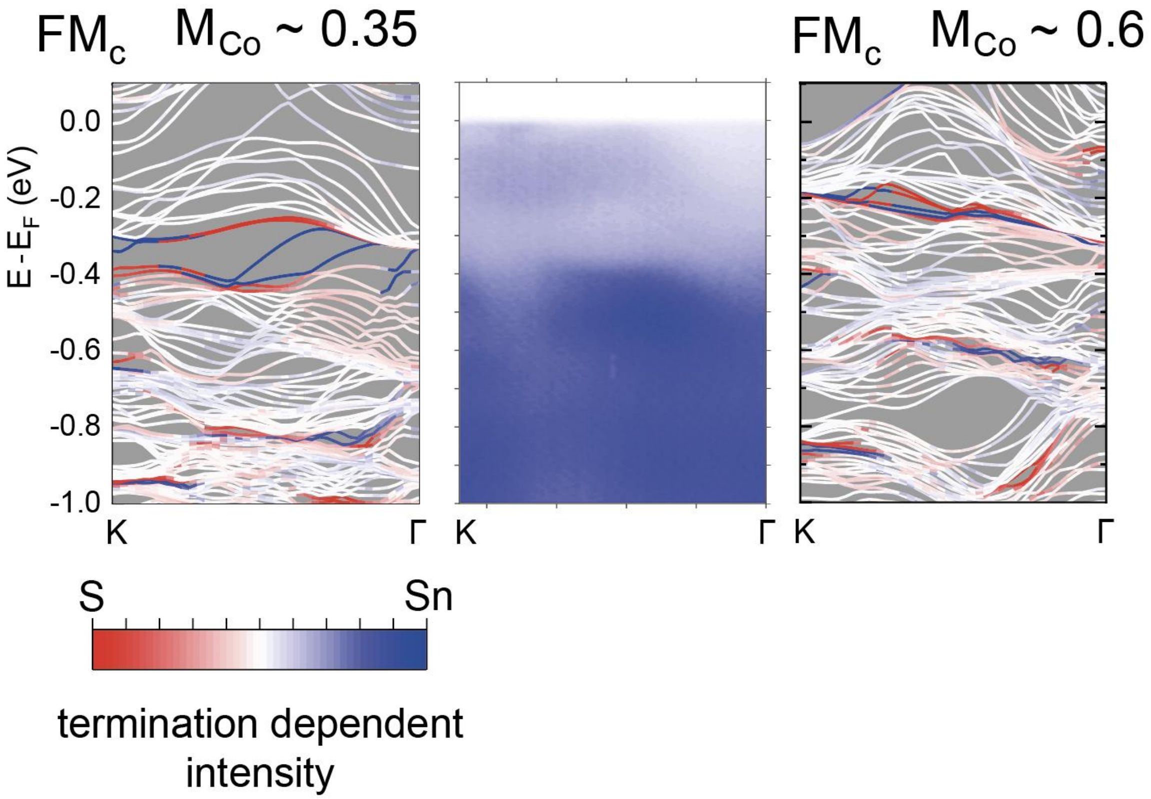

Supplementary Figure 26 Comparison of slab calculation for ferromagnetic phase with cobalt magnetic moment $\mu_{\mathrm{Co}} = 0.35\ \mu_{\mathrm{B}}$ and $\mu_{\mathrm{Co}} = 0.6\ \mu_{\mathrm{B}}$ to ARPES KΓ cut from a synchrotron-based measurement performed at 20 K.
